# Improved Dementia Prediction in Cerebral Small Vessel Disease Using Deep Learning–Derived Diffusion Scalar Maps From T1

**DOI:** 10.1161/STROKEAHA.124.047449

**Published:** 2024-08-15

**Authors:** Yutong Chen, Daniel Tozer, Rui Li, Hao Li, Anil Tuladhar, Frank Erik De Leeuw, Hugh S. Markus

**Affiliations:** Department of Clinical Neuroscience, Stroke Research Group, University of Cambridge, United Kingdom (Y.C., D.T., R.L., H.S.M.).; Department of Neurology, Radboud University Medical Center, Donders Center for Medical Neurosciences, Nijmegen, the Netherlands (H.L., A.T., F.E.D.L.).

**Keywords:** cerebral small vessel diseases, deep learning, diffusion tensor imaging, magnetic resonance imaging, stroke, lacunar

## Abstract

**BACKGROUND:**

Cerebral small vessel disease is the most common pathology underlying vascular dementia. In small vessel disease, diffusion tensor imaging is more sensitive to white matter damage and better predicts dementia risk than conventional magnetic resonance imaging sequences, such as T1 and fluid attenuation inversion recovery, but diffusion tensor imaging takes longer to acquire and is not routinely available in clinical practice. As diffusion tensor imaging–derived scalar maps—fractional anisotropy (FA) and mean diffusivity (MD)—are frequently used in clinical settings, one solution is to synthesize FA/MD from T1 images.

**METHODS:**

We developed a deep learning model to synthesize FA/MD from T1. The training data set consisted of 4998 participants with the highest white matter hyperintensity volumes in the UK Biobank. Four external validations data sets with small vessel disease were included: SCANS (St George’s Cognition and Neuroimaging in Stroke; n=120), RUN DMC (Radboud University Nijmegen Diffusion Tensor and Magnetic Resonance Imaging Cohort; n=502), PRESERVE (Blood Pressure in Established Cerebral Small Vessel Disease; n=105), and NETWORKS (n=26), along with 1000 normal controls from the UK Biobank.

**RESULTS:**

The synthetic maps resembled ground-truth maps (structural similarity index >0.89 for MD maps and >0.80 for FA maps across all external validation data sets except for SCANS). The prediction accuracy of dementia using whole-brain median MD from the synthetic maps is comparable to the ground truth (SCANS ground-truth c-index, 0.822 and synthetic, 0.821; RUN DMC ground truth, 0.816 and synthetic, 0.812) and better than white matter hyperintensity volume (SCANS, 0.534; RUN DMC, 0.710).

**CONCLUSIONS:**

We have developed a fast and generalizable method to synthesize FA/MD maps from T1 to improve the prediction accuracy of dementia in small vessel disease when diffusion tensor imaging data have not been acquired.

Cerebral small vessel disease (SVD) accounts for a quarter of all strokes and is the most common pathology underlying vascular cognitive impairment and dementia.^1^ Characteristic appearances on magnetic resonance imaging (MRI) include lacunar infarcts, white matter hyperintensities (WMHs), and cerebral microbleeds. The extent of WMH correlates with the degree of cognitive impairment and predicts future stroke and dementia.^2^ However, stronger correlations with cognition have been found using diffusion tensor imaging (DTI), which is more sensitive to ultrastructural damages in the white matter. DTI can reveal abnormalities not only in WMH regions but also in normal-appearing white matter, which strongly correlates with cognition and predicts future dementia.^3,4^ DTI-derived metrics have been proposed as surrogate end points to monitor therapeutic intervention in clinical trials in SVD.^3^

However, DTI takes longer to acquire and is not routinely available in clinical practice. As DTI-derived scalar maps—fractional anisotropy (FA) and mean diffusivity (MD)—are widely used in clinical settings,^5^ one approach is to synthesize FA/MD from conventional MRI, such as T1 and fluid attenuation inversion recovery (FLAIR) sequences. This has now become possible with deep learning, as T1 and FLAIR share structural similarities with FA/MD maps.^6,7^ Deep learning models can detect subtle and intricate features too nuanced for human perception^8^ and learn the complex mapping from T1 and FLAIR to FA/MD maps. Several models have been proposed to synthesize FA/MD maps from T1 images.^6,7^ However, these models have been developed on healthy participants or patients with Alzheimer disease and, to date, have only been evaluated in moderate sample sizes while lacking external validation to show generalizability. It is uncertain whether these models can capture FA/MD changes in SVD and whether the synthetic maps predict cognitive outcomes as accurately as ground-truth maps.

To synthesize FA/MD maps from T1 images in SVD, we developed the diffusion scalar generative adversarial network (DS-GAN). DS-GAN is based on generative adversarial networks,^9^ which is well suited for synthesizing images of 1 type of contrast such as FA/MD from another such as T1.^10,11^ We evaluated how the synthetic maps correlated with the ground truth and how they correlated with cognition and predicted dementia. To evaluate generalizability, we developed the model on a cohort with SVD and then tested its performance on 4 independent SVD cohorts.

## METHODS

### Cohort Selection

#### Data Availability

The data sets may be shared with researchers upon reasonable request to the corresponding author and after permission from the regulatory authorities. The UK Biobank (UKB) data are available at www.ukbiobank.ac.uk/register-apply. Study protocols were not prepared.

#### Training Cohort

The training cohort for DS-GAN was obtained from the UKB, a longitudinal cohort study of 100 000 predominantly healthy individuals under the application number 36509^12^; 4998 participants with the highest WMH volumes were selected (Figure S1). Participants without T1, FLAIR, or DTI were excluded. This cohort is named the UKB_WMH cohort and was split into training and internal validation sets in a 9:1 ratio.

#### Validation Cohort

The model was validated on 4 independent cohorts of patients with symptomatic SVD.

1. SCANS (St George’s Cognition and Neuroimaging in Stroke): 121 participants with severe symptomatic SVD defined as a symptomatic lacunar infarct with confluent WMH (Fazekas grade ≥2).^13^
2. RUN DMC (Radboud University Nijmegen Diffusion Tensor and MRI Cohort) study: 503 participants with predominantly mild symptomatic SVD defined as the presence of lacunes and any WMH on neuroimaging and accompanying stroke, subacute cognitive, or motor symptoms.^14^
3. How intensively should we treat PRESERVE (Blood Pressure in Established Cerebral Small Vessel Disease): multicenter clinical trial including 111 participants with severe symptomatic SVD defined as a symptomatic lacunar infarct with confluent WMH (Fazekas grade ≥2).^15^
4. NETWORKS: 26 participants with severe symptomatic SVD.^16^ The inclusion criteria were symptomatic lacunar infarct with confluent WMH (Fazekas grade ≥2). Fourteen of the participants underwent repeated imaging approximately 2 weeks after the first imaging. Due to the small sample size, all results pertaining to the NETWORKS study are shown in the tables in the Supplemental Material.

To test the generalizability of DS-GAN in healthy participants, 1000 participants with the smallest WMH volumes were selected from the UKB. This is named the subset of patients within the UKB that contains the lowest WMH lesions (UKB_normal) data set.

To investigate the cross-sectional association between baseline MRI metrics and cognition, we examined 3 cognitive measures (global cognition, executive functioning, and processing speed). Cognitive scores were determined as *Z* scores using published normative data (Table S1), except in the NETWORKS cohort where an associated control cohort was used to normalize the data.

Two SVD cohorts (SCANS and RUN DMC) provided both cross-sectional and prospective longitudinal data with follow-up (5 years in SCANS and 14 years in RUN DMC), which allowed us to not only examine correlations between baseline MRI and cognition but also to determine whether baseline MRI parameters predicted future dementia.

MRI acquisition parameters are described in Tables S2 through S4. Briefly, all T1 images were acquired as 3-dimensional at a resolution of 1×1×1 mm. FLAIR images were acquired as 3-dimensional in UKB and NETWORKS and as 2-dimensional in SCANS, PRESERVE, and RUN DMC.

### MRI Preprocessing

For UKB, the image preprocessing pipeline has been described.^12^ For the external validation data set, image preprocessing followed a similar pipeline described in Figure S2 and detailed in Section 1.1 in the Supplemental Methods. Briefly, for T1 images, Gibbs artifacts were removed,^17^ magnetic field inhomogeneity was corrected,^18^ and skullstripping^19^ and tissue segmentation were performed. For DTI images, eddy correction^20^ and diffusion tensor fitting were performed to yield FA/MD maps. FLAIR, MD, and FA were rigidly registered to T1.^21^ All images were rigidly registered to the Montreal Neurological Institute space.

T1 and FLAIR images were used for WMH segmentation using the hypermapper package,^22^ except for UKB, where WMH was segmented using BIANCA on FLAIR images.^23^

In all cohorts, the total brain volume (TBV) was calculated using SIENAX^24^ by summing the volumes of the gray matter, white matter, and ventricles and normalizing the total volume by the skull size. The number of lacunes was manually counted by experienced radiologists.

### Deep Learning

**Figure 1.**
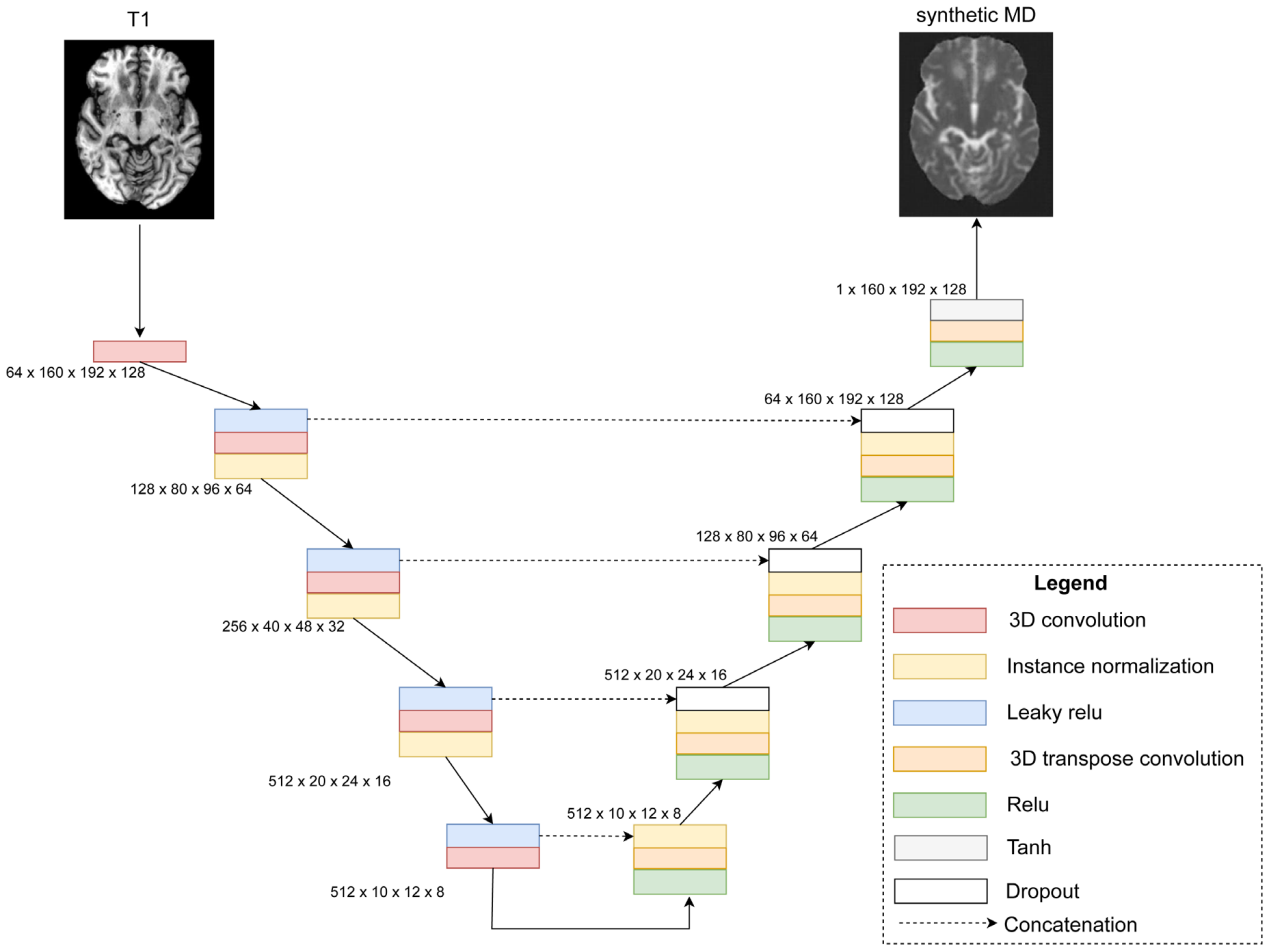
**Diffusion scalar generative adversarial network (DS-GAN) generator structure.** The dimensions of the intermediary outputs are shown as the number of channels×image height×image width×image depth. The diagram shows DS-GAN synthesizing mean diffusivity (MD) maps, which can also be used to synthesize fractional anisotropy maps (not shown). Relu indicates rectified linear unit.

DS-GAN consists of 2 deep learning models: a generator and a discriminator. The generator synthesizes an FA or MD map using T1 and FLAIR images. The goal of the discriminator is to classify the map from the generator as fake and the ground-truth map as real. The goal of the generator is the opposite: to generate realistic maps so that the discriminator cannot tell the difference between fake from real maps. The competing goals between the generator and the discriminator allow both models to improve over time. In this study, the generator was built upon a 3-dimensional U-Net architecture (Figure 1), and the discriminator was the 3-dimensional extension of PatchGAN.^10^

The following hyperparameters were used: epoch number, 60; learning rate, 0.0001; batch size, 1; and Adam optimizer with β0 of 0.5 and β1 of 0.999. To augment the training data set, in each training epoch, each image was randomly translated and downsampled, and random brightness and blurring were applied. The details of the image augmentation, network architecture, and hyperparameter selections are given in Sections 1.2 to 1.4 in the Supplemental Methods.

### Statistics

#### Cohort Characteristics

The distribution of continuous variables was evaluated using the Shapiro-Wilk test. Descriptive statistics, including mean and SD for normally distributed variables or median and interquartile range (IQR) for nonnormally distributed variables, were reported. Demographic variables among ≥3 data sets were compared using 1-way ANOVA for normally distributed data, the Kruskal-Wallis test for nonnormally distributed data, or the χ^2^ test with Yates correction for categorical data. For comparisons between 2 data sets, the *t* test was used for normally distributed data, the Wilcoxon rank-sum test for nonnormally distributed data, and the χ^2^ test with Yates correction for categorical data.

#### Comparison Between Synthetic and Ground-Truth Maps

Three metrics were used to evaluate the similarity between the synthetic FA/MD maps and the ground truth: peak signal-to-noise ratio,^25^ root mean squared error, and structural similarity index measure (SSIM).^26^

For each validation data set, 10% of synthetic MD maps were randomly selected for visual evaluation against the ground truth by an independent researcher in terms of the presence of artifacts, contrast between the normal-appearing white matter (NAWM) and WMH region, presence of new structures, absence of existing structures, and sharpness of the structures seen within the maps (evaluation criteria in Table S5). The researcher was not blinded to the source or the data set. All maps were evaluated under a fixed intensity range between 0 and 0.003 mm^2^/s. Due to the small sample size of the NETWORKS cohort, 6 maps were selected.

To investigate the cause for the errors in the synthetic maps, Pearson correlation was computed between the SSIM of the synthetic MD maps with the ground truth and 3 different variables: age, WMH volume, and T1-to-DTI registration error, which was defined as voxelwise intensity correlation between T1 and FA.

#### Correlation of Metrics Derived From Synthetic Maps With Ground Truth

Metrics derived from the ground truth and synthetic FA/MD maps include peak width of skeletonized MD^27^ and median FA/MD in the whole brain (gray matter, white matter, sulcal cerebrospinal fluid, and ventricles), all white matter, NAWM, and WMH regions. NAWM area was defined as the white matter mask excluding the WMH lesion area. All calculations were done in the Montreal Neurological Institute space. Pearson correlation was computed for each metric between the synthetic and ground-truth maps.

#### Reproducibility of Synthetic FA-/MD-Derived Metrics

The reproducibility of synthetic FA-/MD-derived metrics was evaluated using the baseline and 2-week follow-up scans, which were available in 14 of the 26 participants from the NETWORKS study. Reproducibility was defined by the Pearson correlation of the metrics obtained between the 2 time points.

#### Correlation With Cognition

Pearson correlation was computed between the 3 cognitive domains and the metrics derived from synthetic and ground-truth FA/MD maps. Patients without cognitive data were excluded (0, 0, 1, and 3 patients in SCANS, RUN DMC, PRESERVE, and NETWORKS cohorts, respectively). For comparisons, correlations were performed with WMH volume and TBV, a marker of brain atrophy. To test the significance of the correlation, linear regression was performed between cognition and different metrics while adjusting for age and sex. *P* values of the slopes were obtained and adjusted by the Benjamini-Hochberg method separately for each cognitive domain per data set.

Causal mediation analysis was performed in the RUN DMC cohort to investigate how imaging markers related to SVD—WMH volume, lacune count, and TBV—mediated the association between FA-/MD-derived metrics and cognitive performance (Section 1.5 in the Supplemental Methods).

#### Prediction of Dementia

Univariate Cox proportional hazard models were constructed to predict the onset of dementia using each metric derived from synthetic and ground-truth FA/MD maps, WMH volumes, and TBV. *P* values for the hazard ratio of each metric were adjusted by the Benjamini-Hochberg method per data set.

### Software

All image analysis and deep learning models were implemented in Python 3.8. All statistical analyses were performed in R, version 4.2.0. All CNN models were run on a Nvidia A100 16-GB GPU using PyTorch, version 1.9.0, CUDA, version 11.2, and cuDNN, version 8.1. The computation time to synthesize 1 MD or FA volume from preprocessed T1 and FLAIR images was 8.5±0.1 s on 3 Intel Xeon CPUs and 32±1 ms on an Nvidia A100 GPU. The source code is published (https://github.com/Yutong441/DS-GAN). This article follows the TRIPOD+AI reporting guideline.^28^

### Ethical Statement

The SCANS study received ethical approval from the London–Wandsworth ethics committee (ukctg.nihr.ac.uk; study ID: 4577). The RUN DMC study received ethical approval from the Medical Review Ethics Committee Region Arnhem-Nijmegen. The PRESERVE study received ethical approval from the Harrow National Research Ethics Service Committee (REC number: 11/LO/0458) and is registered with the UK Clinical Research Network (CRN number: 10962). The NETWORKS study received ethical approval from the East of England-Cambridge East Research Ethics Committee (reference: 14/EE/0014). All participants provided written informed consent according to the Declaration of Helsinki.

## RESULTS

### Cohort Characteristics

After excluding participants without DTI, T1, or FLAIR images, 4998, 1000, 120, 502, 105, and 26 participants remained in the UKB_WMH, UKB_normal, SCANS, RUN DMC, PRESERVE, and NETWORKS data sets, respectively (Figure S1). Compared with UKB_normal, participants in the UKB_WMH cohort are older (71 [IQR, 67.0–74.0] versus 55 [IQR, 52.0–60.0] years; *P*<0.001), less likely to be female (44% versus 60%; *P*<0.001), and have higher WMH volumes (15.5 [IQR, 12.3–21.9] versus 0.3 [IQR, 0.3–0.4] mL; *P*<0.001; Table 1). Across the external validation data sets, participants in the RUN DMC cohort are younger (64.8 [IQR, 58.1–73.0] versus 69.8 [IQR, 63.7–75.6] years; *P*<0.001) and have smaller WMH volumes (2.7 [IQR, 1.0–7.7] versus 24.1 [IQR, 13.2–39.8] mL; *P*<0.001) than the other validation data sets.

**Table 1.**
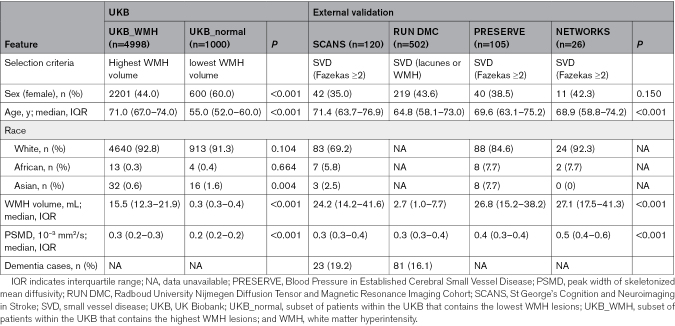
Baseline Demographics

### Development of DS-GAN

We tested the performance of DS-GAN in synthesizing FA/MD from different input MRI sequences: T1 and FLAIR, T1 only, and FLAIR only. In all external validation sets except UKB_normal, the model using T1 as the sole input achieved the highest performance in most performance metrics (Table S6). The SSIM of the synthetic maps was >0.89 for MD maps and >0.80 for FA maps across all external validation data sets except for SCANS (SSIM=0.818 for MD and 0.766 for FA). The model using FLAIR as the sole input achieved the lowest performance. Therefore, in subsequent analyses, we used the model using T1 as the sole input. The performance of the model using T1 as the sole input is shown in Table 2.

**Table 2.**
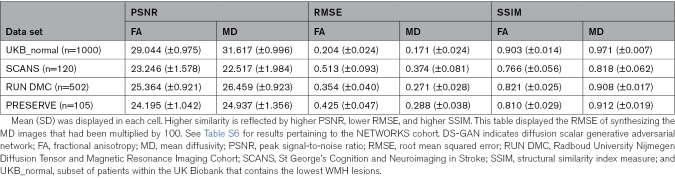
Comparison of FA/MD Maps Between the Ground Truth and Those Synthesized by DS-GAN, Using PSNR, RMSE, and SSIM

### Comparison Between Synthetic and Ground-Truth Maps

DS-GAN successfully synthesized FA/MD maps from T1 in the validation cohorts. The synthetic FA-/MD-derived metrics were highly correlated with ground truth (for whole-brain median MD; R=0.927 [SCANS], 0.907 [RUN DMC], 0.818 [PRESERVE], and 0.757 [UKB_normal]). From the synthetic maps, whole-brain median FA/MD demonstrated higher correlations with the ground truth than median FA/MD in WMH regions and NAWM (Table S7).

The performance of DS-GAN, as assessed by peak signal-to-noise ratio, SSIM, and root mean squared error, was similar in PRESERVE and RUN DMC (Table 2), but the accuracy of synthesis was lower in SCANS. Performance was highest in normal control (UKB_normal). Variation in the synthesis performance could not be explained by age (R^2^<0.1 across all data sets) or WMH volume (R^2^<0.1 across all data sets). The synthesis performance was correlated with registration errors (R^2^>0.3 across all data sets; Figure S4).

**Figure 2.**
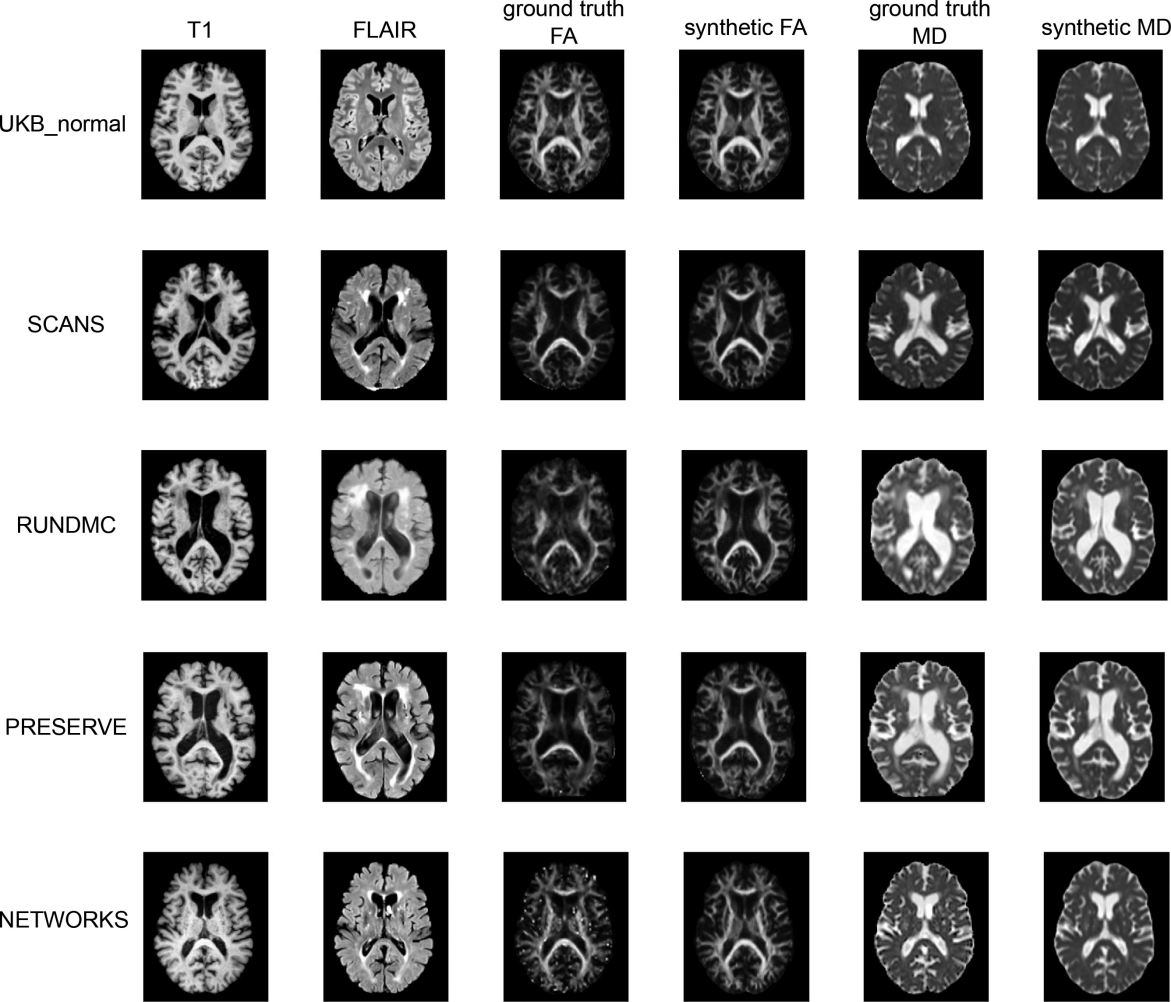
**Examples of ground-truth and synthetic fractional anisotropy (FA)/mean diffusivity (MD) maps from 5 validation data sets.** FLAIR indicates fluid attenuation inversion recovery; PRESERVE, Blood Pressure in Established Cerebral Small Vessel Disease; SCANS, St George’s Cognition and Neuroimaging in Stroke; and UKB_normal, a subset of patients within the UK Biobank that contains the lowest white matter hyperintensity lesions.

Synthetic FA/MD maps resembled the ground truth (eg, shown in Figure 2). The white matter tracts were clearly visualized on the synthetic FA maps (Figure 2) although in the magnified view of the internal capsule, the synthetic maps appeared smoothened with fewer fine structural details (Figure S3). In visual evaluation, synthetic maps neither created new structures nor missed existing structures in any maps (Table S8). However, they exhibited moderately higher levels of artifacts (in 8%, 8%, and 45% of a selection of the SCANS, RUN DMC, and PRESERVE cohorts, respectively), moderately lower levels of contrast between WMH and NAWM (8%, 0%, and 36%), and moderately lower sharpness in SCANS and PRESERVE (58% and 27%). In RUN DMC, 83% of the evaluated maps have moderately higher levels of sharpness compared with the ground truth.

### Reproducibility of Synthetic FA-/MD-Derived Metrics

Comparing repeated MRI scans in the NETWORKS study showed a high level of reproducibility in synthetic FA-/MD-derived metrics (correlations ranging between 0.927 and 0.996; Table S9). For the synthetic FA/MD maps, most metrics displayed higher reproducibility than the ground truth, whereas median MD within NAWM showed lower reproducibility.

### Correlation With Cognition

Synthetic FA-/MD-derived metrics correlated with cognition at baseline almost, as well as the ground truth, and better than WMH lesion volume and TBV. For example, for whole-brain median MD, correlations with global cognition are given as follows: in SCANS, ground truth, −0.450 (*P*<0.001); synthetic, −0.410 (*P*<0.001); WMH volume, −0.138 (*P*=0.069); and TBV, 0.240 (*P*=0.029); in RUN DMC, ground truth, −0.518 (*P*<0.001); synthetic, −0.463 (*P*<0.001); WMH volume, −0.278 (*P*=0.005); and TBV, 0.330 (*P*=0.002). Results of correlations for all metrics are shown for global cognition in Table 3, for executive function and processing speed in Tables S10 and S11, and separately for the NETWORKS cohort in Table S12.

**Table 3.**
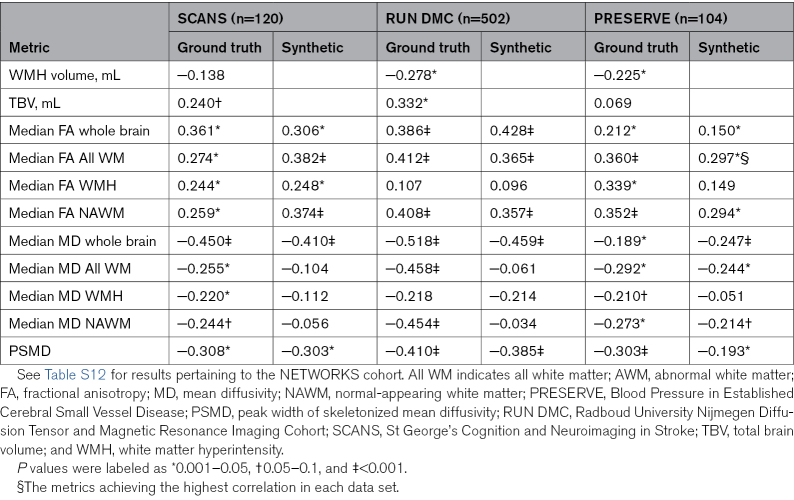
Correlation of the Metrics Derived From Synthetic FA/MD and the Ground-Truth Metrics With Global Cognition

We investigated how SVD imaging markers (WMH volume, lacunes, and TBV) mediated the association between cognition and the whole-brain median FA/MD. Between ground truth versus the synthetic maps, SVD imaging markers did not have significantly different effects on the association between cognition and whole-brain median FA/MD (Table S13; Section 1.2 in the Supplemental Results).

### Prediction of Dementia

The synthetic FA-/MD-derived metrics predicted dementia to a similar level to that found with the ground truth. The c-index for prediction by whole-brain median MD was similar in SCANS (ground truth, 0.822; synthetic, 0.821) and in RUN DMC (ground truth, 0.816; synthetic, 0.812; Table 4). The performance of synthetic whole-brain MD in predicting dementia was higher than that for WMH volume (SCANS, 0.534; RUN DMC, 0.710) and TBV (SCANS, 0.709; RUN DMC, 0.739). The accuracy of predicting dementia was minimally improved by incorporating 3 demographic factors (SCANS, 0.828; RUN DMC, 0.845; Table S14) and was more highly improved by incorporating 3 cognitive scores (executive function, processing speed, and global cognition; SCANS, 0.903; RUN DMC, 0.858; Table S15).

**Table 4.**
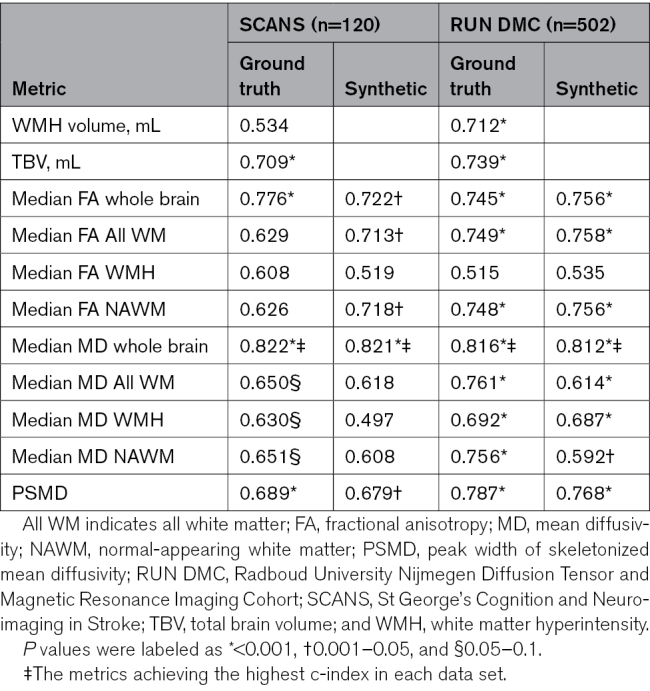
C-Index in Predicting Dementia Onset by Different Metrics in Univariate Cox Proportional Hazard Models

## DISCUSSION

We have demonstrated that using deep learning, it is possible to synthesize FA/MD maps from T1 images in patients with SVD, and synthetic maps predict future dementia almost as accurately as the ground-truth maps and better than WMH lesion volume. Although the synthetic maps themselves seem to be less sharp and oversmoothened and have not reached the point of replacing DTI in clinical settings, the metrics obtained from these maps correlated well with metrics from the ground-truth FA/MD maps and were shown to be reproducible in a cohort with repeat scans. Future studies could evaluate the use of these metrics in clinical trials.

Our study has many strengths. This is the first application of deep learning to synthesize FA/MD maps in patients with SVD. We used a large training sample (n=4998). Performance was consistent across 4 independent validation cohorts encompassing a wide range of SVD severity. The model generalizability was corroborated by the high performance in normal controls without SVD (SSIM, 0.971±0.007 for MD and 0.903±0.014 for FA, respectively). This matched the values reported by previous studies in patients with Alzheimer disease (SSIM, 0.963±0.009 for MD and 0.959±0.007 for FA)^7^ and healthy participants (0.937 for MD and 0.861 for FA).^7^

However, the model also has limitations. The median MD in NAWM is not as accurate compared with whole-brain values. This could be attributed to the lack of sensitivity of T1 images in capturing the subtle changes in NAWM. Contrastingly, the WMH regions display higher contrast in MD, which could be more easily captured by DS-GAN. This inaccuracy in MD calculation in NAWM could explain the low correlation of the associated synthetic metrics with cognitive performance. Thus, the ability to investigate MD changes in NAWM is reduced compared with DTI-derived FA/MD maps.

Second, it is unclear whether FA/MD synthesis could be generalized to other neurological conditions. As the testing samples of DS-GAN mainly consisted of participants with SVD, DS-GAN was not assessed in patients with larger nonlacunar infarcts, tumors, or demyelinating lesions such as multiple sclerosis. It is unclear how these other pathologies would be reflected in the synthetic FA/MD maps.

Third, the performance of DS-GAN on SCANS was lower, possibly because of the poor registration between the T1 and the FA/MD maps. This misalignment confounds the evaluation of the voxelwise similarity between the ground-truth and synthetic maps.

Fourth, combining T1 and FLAIR images into DS-GAN did not improve model performance compared with only using T1. This could be because FLAIR did not convey additional structural information beyond that in T1. Minor structural misalignments between T1 and the coregistered FLAIR images could also limit the accuracy of synthesis. The accuracy of synthesizing FA/MD was lower in the model using only FLAIR images compared with that using only T1 images. This could be because the FLAIR images in the validation data sets were of lower resolution along the axial slices compared with T1. This could limit the application of DS-GAN to high-resolution T1 images.

Fifth, dementia prediction using synthetic FA/MD maps was validated in 2 of the 5 external validation data sets that contain follow-up information on the dementia status. Future studies should evaluate the dementia prediction value of the synthetic maps in more data sets.

Finally, from the standpoint of rapid acquisition of diffusion scalar maps that correlate with cognition, diffusion-weighted imaging, which is faster to acquire than DTI, can generate apparent diffusion coefficient maps that correlate with cognitive outcomes.^29^ However, compared with diffusion-weighted imaging, the advantage of DS-GAN in synthesizing FA/MD from T1 is retrospective FA/MD synthesis in old data sets where diffusion-weighted imaging or DTI is unavailable, such as DNA lacunar.^30^ Also, future studies could extend DS-GAN to synthesize other diffusion MRI scalar maps such as orientation dispersion index and isotropic volume fraction.

In conclusion, DS-GAN is a fast, reproducible, and generalizable deep learning model that can synthesize FA/MD maps from T1 images. These synthetic metrics correlate with ground truth and predict dementia in patients with SVD almost, as well as ground-truth FA/MD maps, and better than WMH lesion volume, the most widely used SVD-related clinical imaging marker. The model offers a quick and cost-effective way to estimate FA/MD-based metrics from conventional MRI sequences.

## ARTICLE INFORMATION

### Acknowledgments

Y. Chen, Dr Tozer, and H.S. Markus conceived the study. H.S. Markus provided the SCANS (St George’s Cognition and Neuroimaging in Stroke), PRESERVE (Blood Pressure in Established Cerebral Small Vessel Disease), and NETWORKS data sets. Dr De Leeuw provided the RUN DMC (Radboud University Nijmegen Diffusion Tensor and Magnetic Resonance Imaging Cohort) data set. Y. Chen, Dr Tozer, R. Li, and Dr Li were involved in preprocessing the T1, fluid attenuation inversion recovery, and diffusion tensor imaging images. Y. Chen constructed the deep learning model and performed statistical analysis. Y. Chen and H.S. Markus drafted the article. All authors were involved in article revision. No patients were involved.

### Sources of Funding

This research was funded by a British Heart Foundation Programme grant (RG/F/22/110052). Infrastructural support was provided by the Cambridge British Heart Foundation Centre of Research Excellence (grant RE/18/1/34212) and by the Cambridge University Hospitals National Institute for Health and Care Research (NIHR) Biomedical Research Centre (grant NIHR203312). The views expressed are those of the authors and not necessarily those of the NIHR or the Department of Health and Social Care.

### Disclosures

None.

### Supplemental Material

Supplemental Methods

Supplemental Results

Tables S1–S16

Figures S1–S6

References 31–49

## Supplementary Material


