# Supplementary material for: Improved Dementia Prediction in Cerebral Small Vessel Disease Using Deep Learning–Derived Diffusion Scalar Maps From T1
Source: Stroke. 2024 Aug 15;55(9):2254–63. doi: 10.1161/STROKEAHA.124.047449 (PMC11346716; doi:10.1161/STROKEAHA.124.047449)
Supplement: Supplementary file 1 [file str-55-2254-s001.pdf]

SUPPLEMENTAL MATERIAL

Yutong Chen[1], Daniel Tozer[1], Rui Li[1], Hao, Li[2], Anil Tuladhar[2], Frank Erik De Leeuw[2] and Hugh S Markus[1]

1 Stroke Research Group, University of Cambridge, Cambridge, UK

2 Radboud University Medical Center, Department of Neurology; Donders Center for Medical Neurosciences, 6500 HB Nijmegen, The Netherlands

1 Supplemental Methods

1.1 MRI preprocessing

1.1.1 UKB

Preprocessed imaging data was downloaded as NiFTI files from UKB. The data preprocessing pipeline has been described.[12] Skullstripped T1 and FLAIR images without bias correction were downloaded. In both T1 and FLAIR images, Gibbs ringing artifacts were removed using the mrdegibbs function from the mrtrix package,[17] and magnetic field non-homogeneity was corrected by the N4BiasCorrection function from the ANTs package.[18] FLAIR images were registered to T1 images via rigid registration using the ANTs package. Preprocessed FA and MD images were downloaded. The mean b0 volumes were extracted from the eddy corrected DTI data. The mean b0 volumes were registered to T1 via rigid registration using the epi_reg function from the FSL package.[21] The resulting transformation matrix was used to register FA and MD to T1. T1 was registered to the Montreal Neurological Institute (MNI) template from FSL via rigid registration. The resulting transformation matrix was used to warp FLAIR, FA and MD–all of which in T1 space–to the MNI space.

1.1.2 SCANS

For T1 and FLAIR images, Gibbs artifacts were removed using the mrtrix package and magnetic field non-homogeneity was corrected using the ANTs package. T1 images were skullstripped using the HD-BET package.[19] Tissue segmentation was performed in skullstripped T1 images using FSL.

The DTI data contained volumes that have been acquired under 25 directions, and 25 diametrically opposite directions to enable removal of cross terms in the signal. Motion correction was performed using the eddy_correct function from FSL. Skullstripping on the b0 volume was performed using FSL brain extraction tool. Diffusion tensor fitting was performed on the DTI data using FSL, which outputs the FA and MD images.

To transform all images to the MNI space, FLAIR images were registered to T1 images via rigid registration. The mean b0 volumes were registered to T1 via rigid registration using the epi_reg function from the FSL package. The resulting transformation matrix was used to register FA and MD to T1. T1 was registered to the MNI152 template from FSL via rigid registration. The resulting transformation matrix was used to warp FLAIR, FA and MD–all of which in T1 space–to the MNI space (Figure S2).

1.1.3 RUNDMC

T1 and FLAIR image processing followed the same pipeline as SCANS. DTI images were denoised using the dwidenoise function from the mrtrix package, and Gibbs artifacts were removed. Motion correction was performed on the DTI images using the mcflirt function from FSL. The b0 volumes were averaged. The mean b0 volume and the corresponding skullstripped T1 image were used to synthesise the reverse phase encoding DTI image.[20] This allowed for topup and eddy correction using FSL. Diffusion tensor fitting and registration to MNI space followed the same pipeline as SCANS.

1.1.4 PRESERVE

The PRESERVE dataset was processed using the same pipeline as the RUNDMC dataset.

1.1.5 NETWORKS

T1 and FLAIR preprocessing in NETWORKS followed the same pipeline as the SCANS dataset. The DTI data contained minimal susceptibility-induced distortions from visual inspection (Figure S5). After performing eddy correction, the degree of registration of FA with T1, as measured by the Pearson correlation, reduced from a median value of 0.849 (interquartile range [IQR] 0.837-0.858) to 0.841 (IQR 0.801-0.856) (P<0.001, Wilcoxon signed rank test). Only motion correction was applied using the eddy_correct function from FSL. Diffusion tensor fitting and registration to MNI space followed the same pipeline.

1.2 Input to the deep learning network

The backgrounds of all images were cropped such that each image has the dimension of 160×192×128 voxels. Within the brain mask generated by skullstripping, voxel intensity was standardised by z-transform for T1 and FLAIR images. The z-scores were capped to between -2 and +2, and then the score was divided by 2 to confine the intensity values between -1 and 1.

To augment the training dataset, in each training epoch, each 3D volume was translated along the x-y plane randomly between zero and ten voxels and downsampled by a random scale between 0% to 5%. The brightness was randomly altered between 0 and 10%. Random blurring was employed using a Gaussian filter of a randomly selected standard deviation between zero and three voxels. Images were randomly shuffled during training. Whenever model performance was evaluated, the original images without random alteration were used.

1.3 Network architecture

In the DS-GAN model, the generator consists of five convolutional operations and five transpose convolutions. Each convolution had a stride of two voxels, kernel size of four voxels, and was followed by an instance normalization layer, and a leaky relu operation with 0.2 for the negative slope. Each transpose convolution had a stride of two voxels, kernel size of four voxels and was followed by instance normalization and relu. Dropout of 50% of the weights was added between instance normalization and relu in the second, third and fourth transpose convolution block. The final transpose convolutional layer was followed by a tanh function, which outputs the synthetic FA/MD map with intensity values between -1 and 1. This intensity range was rescaled to between 0 and 1.

1.4 Hyperparameter selection

During training, the learning rate was 0.0001 for the first 40 epochs, and decayed linearly to 0 over the following 20 epochs (Supplemental results and Figure S6). The batch size was 1. The loss function was based on the conditional GAN loss functions: least-squared GAN loss plus L1 distance between the output and ground truth. Normalized root mean squared error (NRMSE) loss was added to the base loss function. Three weights of NRMSE loss were investigated: 0, 10 and 100. Adam optimizer with β0 of 0.5 and β1 of 0.999 was used.[31]

In addition to 3D models, a 2D version of DS-GAN was built with the 3D convolutional layers being replaced with 2D convolutions to investigate whether the 2D model can improve performance. Within a training epoch, each training batch consists of 4 randomly selected axial slices from a particular subject. Axial slices were randomly chosen across training epochs, to maximize the diversity of training samples.

Feature matching loss[32], spectral normalization[33] and self-attention[34] were explored to improve model performance. Feature matching loss consisted of mean squared error loss of the outputs from each intermediate block in the PatchGAN discriminator. The weight of the loss was set to 1. Spectral normalization was applied to the last layer of the discriminator. Self-attention module was added to the second and fourth convolutional layers of the downsampling arm of the U-Net, and to the third transpose convolutional layer of the upsampling arm of U-Net.

During model selection, models were trained and tested on a random subset of 500 samples from the derivation cohort (UKB_WMH). This subset was randomly split into 400 training subset and the 100 validation subset. The validation subset was used to select the model with the highest performance in peak signal to noise ratio (PSNR) during the training process. Early stopping was not applied. The best model was selected by the PSNR performance on the validation subset.

After obtaining the optimal hyperparameters, three separate models were trained to handle three different inputs: T1 and FLAIR, T1 only, and FLAIR only. They were trained with the full cohort of the UKB_WMH and tested on all of the external validation datasets. The best model on the external validation datasets were selected for subsequent analyses.

1.5 Mediating role of SVD imaging markers

Causal mediation analysis was performed to assess how imaging markers related to SVD—WMH volume, lacune count and TBV—mediated the association between FA/MD-based metrics and cognitive performance. This was carried out in the RUNDMC cohort.

Each analysis was adjusted for age, sex and years of education. Four FA/MD-based metrics were investigated: ground truth whole-brain MD and FA and synthetic whole-brain MD and FA. Standardised indirect effect of SVD imaging marker on the association between FA/MD-based metrics and cognition was used as the strength of mediation. For each

cognitive marker, p-values were adjusted for multiple testing across all 3 SVD imaging markers and 4 FA/MD-based metrics (3 x 4 =12 times) using the Benjamini-Hochberg test. Causal mediation analysis was performed using the CMAverse package in R.

To compare the indirect effect of SVD imaging markers on the association between FA/MD-based metrics and cognition between ground truth and synthetic metrics, we used the statistical test described in a previous study.[35]

2 Supplemental Results

2.1 Hyperparameter selection

During the training of DS-GAN, the SSIM in both the training and validation datasets plateaued at 40th epoch, before the model began to overfit to the training set for the last 20 epochs. This suggests training the model for 60 epochs is sufficient for model convergence.

In optimizing the hyperparameters in the DS-GAN model, the model was trained in synthesizing MD images from T1 and FLAIR in a random subset of 400 patients in the UKB_WMH dataset and validated on 100 patients from the same dataset. From the accuracy of synthesizing MD in terms of PSNR in the validation dataset, the optimal weight of the NMSE contribution to the loss function was 100. Both spectral normalization and feature matching improved model performance in the validation set. Adding self-attention modules did not improve model performance (Table S16).

2.2 Mediating role of SVD imaging markers

We investigated how SVD imaging markers (WMH, lacunes, TBV) mediated the association between cognition and whole-brain median MD/FA. For both ground truth and synthetic metrics, lacune counts significantly mediated the association between cognition and FA/MD-based metrics (e.g., for global cognition, indirect effect coefficients [β] = -0.031 [95% CI -0.051--0.006, P < 0.001] for ground truth median MD, β=-0.032 [95% CI -0.053--0.004, P < 0.001] for synthetic median MD) (Table S13). TBV significantly mediated the association between global cognition and ground truth median FA (β=0.035 [95% CI, 0.010-0.056, P <0.001]) and between global cognition and synthetic median FA (β=0.034 [95% CI, 0.008-0.059, P <0.001]). WMH volume significantly mediated the association between processing speed and both ground truth and synthetic median MD/FA. Between the ground truth and synthetic metrics, SVD imaging markers did not have significantly different effects on the association between cognition and whole-brain median MD/FA (Table S13).

3 Supplemental Figures

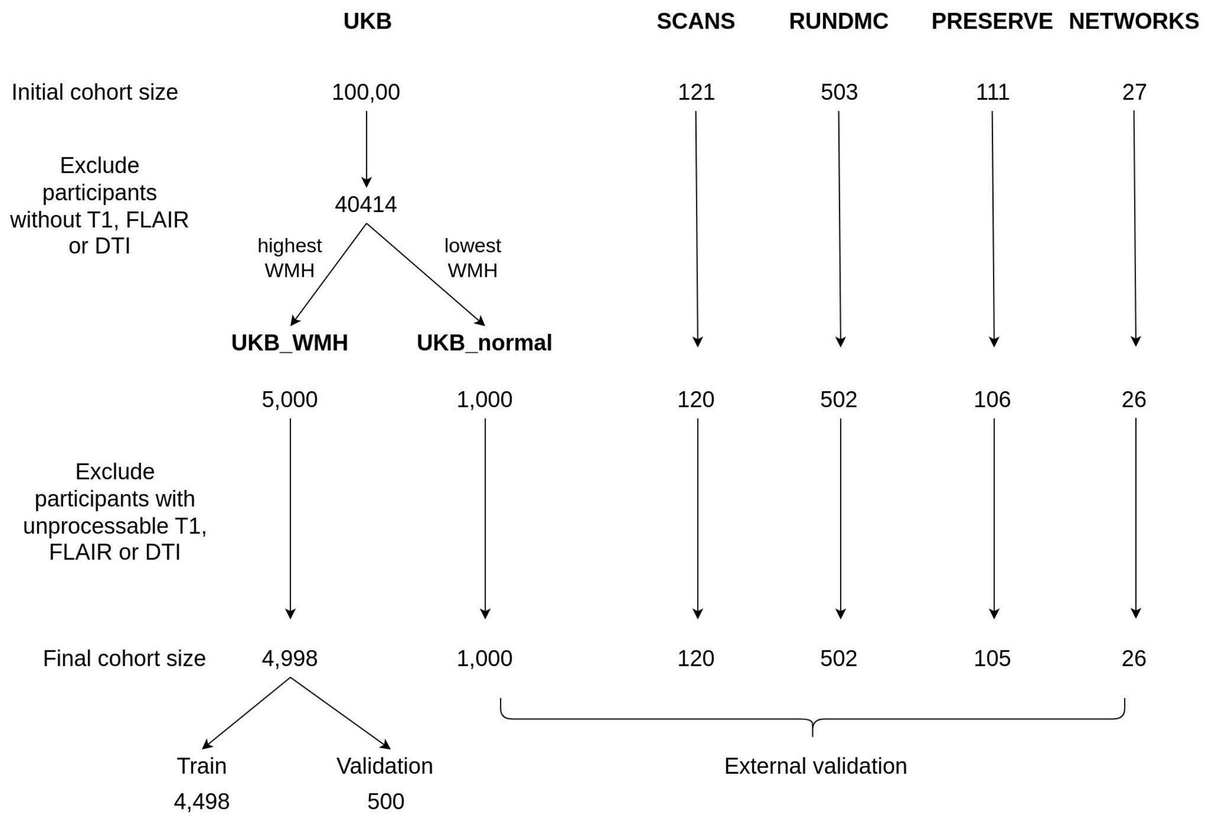

Figure S1 Cohort selection. Abbreviations: WMH: white matter hyperintensity, DTI: diffusion tensor imaging.

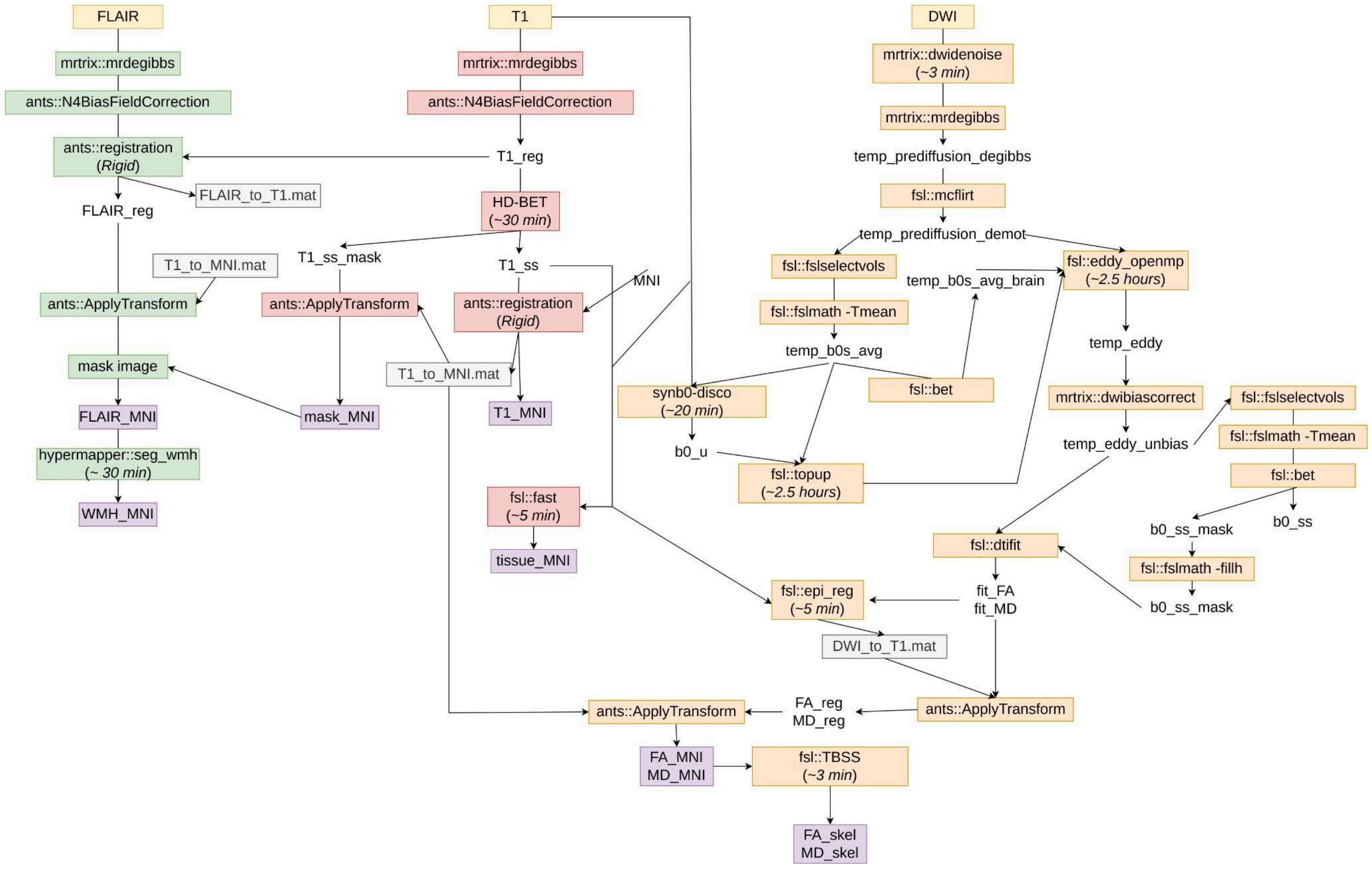

Figure S2 MRI preprocessing pipeline. FLAIR-related preprocessing is colored as green. T1-related preprocessing is colored as red. DWI-related preprocessing is colored as orange. Input images are colored as yellow. Output images are colored as purple. Preprocessing steps are named as ”package name::command name”. Abbreviations: MNI: Montreal Neurological Institute brain template.

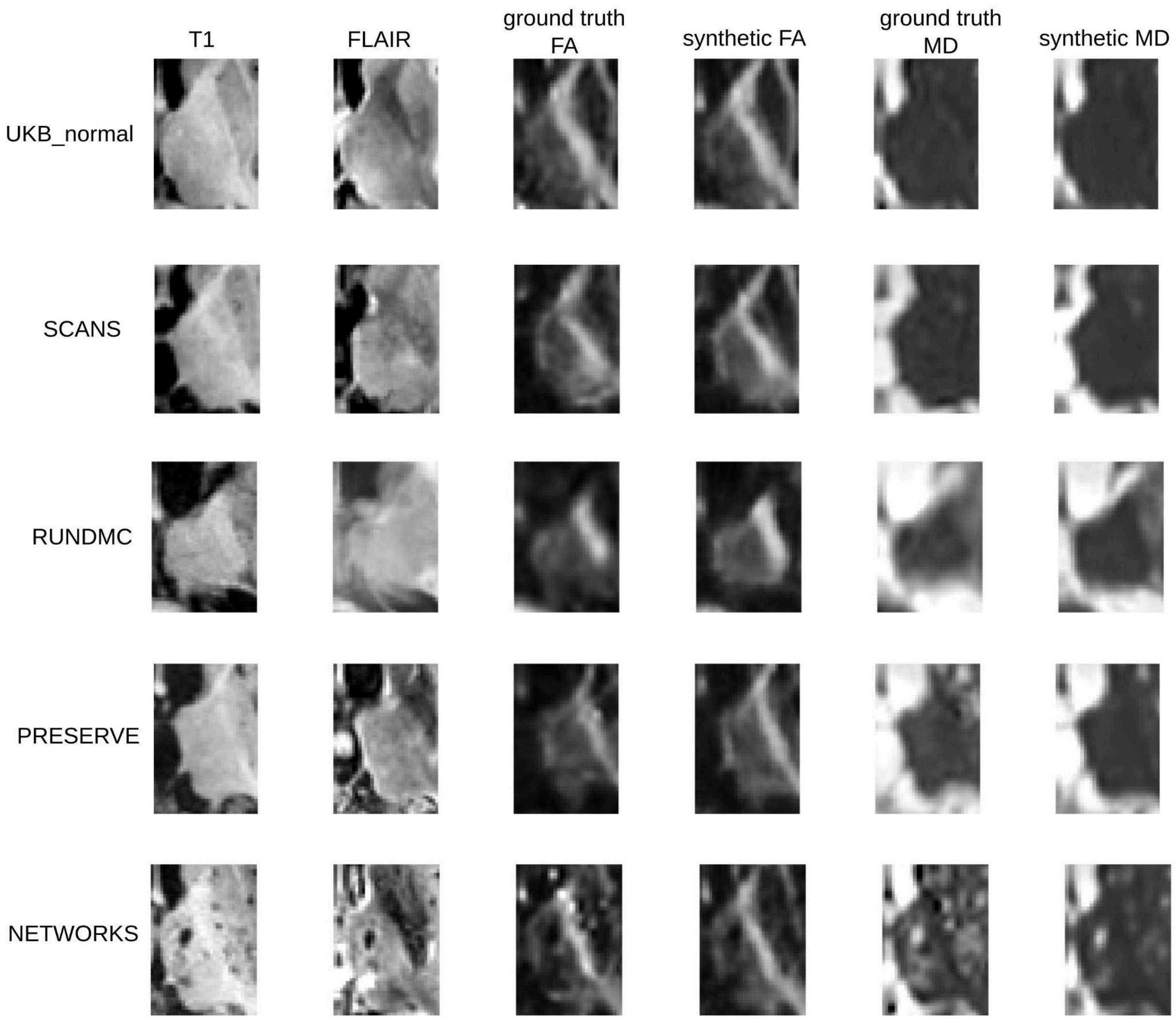

Figure S3 Examples of ground truth and synthetic FA/MD maps from five validation datasets with magnification in the left internal capsule region.

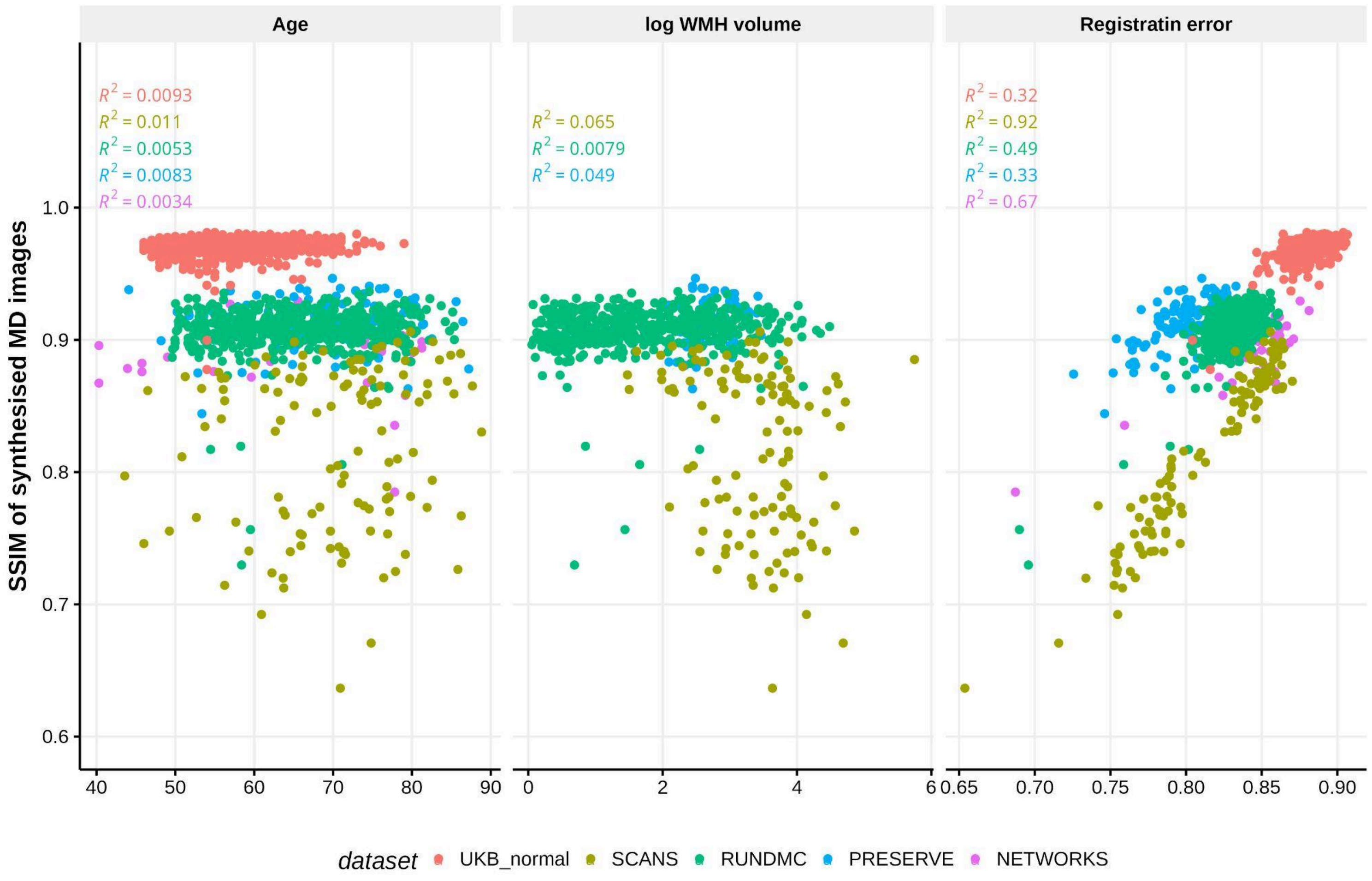

Figure S4 Relationship of structural similarity index measure (SSIM) of the synthetic MD image with registration accuracy, age and log WMH volume. Abbreviations: MD: mean diffusivity, WMH: white matter hyperintensity, DTI: diffusion tensor imaging.

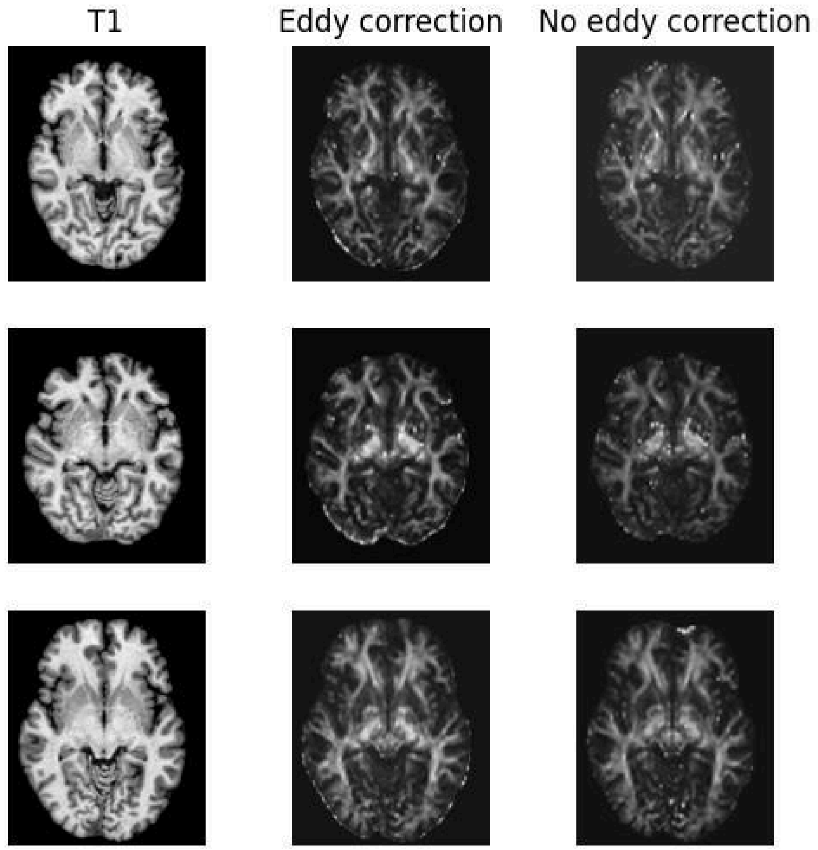

Figure S5 Examples of T1 and FA images in the NETWORKS study. Each row contains the images from one patient.

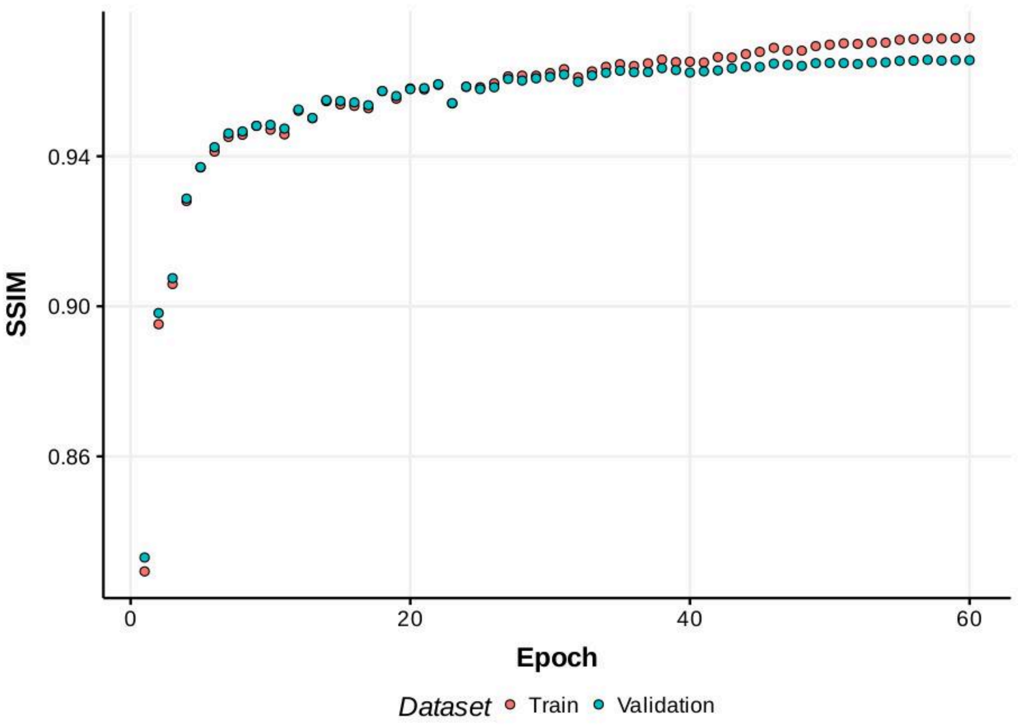

Figure S6 Structural similarity index measure (SSIM) in the training and validation sets during the training of the DS-GAN to synthesise the mean diffusivity (MD) map.

4 Supplemental Tables

Table S1 Cognitive tests used in each study.

Cohort	Variable	Test	Description
SCANS	Executive function	TMT-B	Trail-making test-B, alternating letters and numbers as quickly as possible while still maintaining accuracy[36]
		SL-verbal fluency	Timed generation of words beginning with letter[37]
		mWCST	Modified Wisconsin Card Sorting Test: flexible shifting from learned dimensions[38]
	Processing speed	BMIPB SOIP	Speeded cancellation of second highest of five two-digit numbers[39]
		Digit symbol	Speeded transcoding task[40]
		Grooved pegboard	Pick-up, rotation and placement of small pegs[39]
	Memory	Logical memory	Immediate and delayed recall of short stories[41]
		Visual reproduction	Immediate and delayed reproduction of line drawings[41]
	Global cognition		Combination of executive function, processing speed and long term memory
RUNDMC	Executive function	Inhibition	Divide the Stroop part III SAT score by the mean of the SAT score of parts I and II[42,43]
		VSAT	Verbal Series Attention Test: forward and reverse generation of arithmetic series; number-letter sequencing; auditory vigilance[44]
		Verbal fluency	Naming animals and professions
	Processing speed	Processing speed	
		PPMS	Pencil-pen memory scanning[45]
		Stroop test	Color word test[42,43]
		SDST	Symbol–Digit Substitution Task involving match letters to numbers[46] according to a key
	Memory	RALVT	Rey Auditory Verbal Learning Test[47]
		RCFT	Rey Complex Figure Task involves reproducing a complicated line drawing, first by copying it freehand, and drawing from memory[47]
	Global cognition		Combination of PPMS, Stroop, SDST, RALVT
PRESERVE	Executive function	TMT-B	Trail-making test-B: alternating letters and numbers as quickly as possible while still maintaining accuracy[36]

		Fluency	Naming letters and animals as soon as possible
	Processing speed	WAIS	Wechsler Adult Intelligence Coding test: coding numbers with characters according to a key[40]
		TMT-A	Trail-making test-A: connecting a set of 25 dots as quickly as possible while still maintaining accuracy[36]
	Memory	RALVT	Rey Auditory Verbal Learning Test[47]
	Global cognition		Combine executive function, processing speed and memory
NETWORKS	Executive function	TMT-B	Trail-making test-B: alternating letters and numbers as quickly as possible while still maintaining accuracy[36]
		WCST	Wisconsin Card Sorting Test: flexible shifting from learned dimensions[48]
		Fluency	Naming letters and animals as soon as possible
	Processing speed	TMT-A	Trail-making test-A: connecting a set of 25 dots as quickly as possible while still maintaining accuracy[36]
	Memory	WMS	Wechsler Memory Scale: assess verbal and non-verbal memory[49]
	Global cognition		Combine executive function, processing speed and memory

Abbreviations: SAT: speed accuracy trade-off.

Table S2 MRI scanner details of each cohort.

Cohort	Scanner
UKB	3T Siemens Skyra
SCANS	1.5T General Electric Sigma HDxt
RUNDMC	1.5 Siemens Magnetom Avanto
PRESERVE	8 different 3T scanners
NETWORKS	3T Siemens Magnetom Verio syngo

Table S3 MRI sequence parameters.

Sequence	Parameter	UKB	SCANS	RUNDMC	NETWORKS
T1	TR(ms)	2000	11.5	22.5	2300
	TE(ms)	2.01	5	3.68	2.98
	Slice(mm)	1	1.1	1	1
FLAIR	TR(ms)	5000	9000	9000	12540
	TE(ms)	395	130	84	132
	Slice(mm)	1	5	3	2
DTI	TR(ms)	3600	15600	10200	11700
	TE(ms)	92	93.4	95	106
	Slice(mm)	2	2.5	2	2
	b-values(s/mm^2)	1000, 2000	1000	900	1000
	b0 volumes	5	1	4	4
	RPE volumes	3	0	0	0
	Directions	100	25	61	59

Abbreviations: RPE: reverse phase encodings, TR: repetition time, TE: echo time.

Table S4 PRESERVE MRI sequence parameters across all sites.

Sequence	Parameter	Site 1	Site 2	Site 3	Site 4	Site 5	Site 6
	TR(ms)	8.24	9.81	2200	11	8.53	2200
T1	TE(ms)	4.61	4.60	2.97	4.61	4.61	2.94
	Slice(mm)	1	1	1	1	1	1
	TR(ms)	11000	11000	8000	11000	11000	8000
FLAIR	TE(ms)	120	120	124	120	120	121
	Slice(mm)	3	3	3	3	3	3
	TR(ms)	6850	6850	6850	6850	9100	11500
	TE(ms)	75	75	75	75	82	93
	Slice(mm)	2	2	2	2	2	2
DTI	b-values(s/mm^2)	1000	1000	1000	1000	1000	1000
	b0 volumes	1	1	1	1	1	1
	RPE volumes	0	0	0	0	0	0
	Directions	32	32	32	32	32	32

Table S5 Visual evaluation criteria.

Scores	Description
Artefacts	
0	Same level in ground truth and synthetic maps
1	Moderate differences between synthetic maps and ground truth
2	Major differences between synthetic maps and ground truth
Contrast between normal-appearing white matter and white matter hyperintensity region	
0	Same level in ground truth and synthetic maps
1	Moderate differences between synthetic maps and ground truth
2	Major differences between synthetic maps and ground truth
Presence of new structures	
0	No new structures in synthetic maps
1	Some new structures in synthetic maps
2	Many new structures in synthetic maps
Absence of structures	
0	No missing structures in synthetic maps
1	Some missing structures in synthetic maps
2	Many missing structures in synthetic maps
Visibility and sharpness of white matter hyperintensity	
0	Same visibility and sharpness in GT and S
1	Moderate differences between synthetic maps and ground truth
2	Major differences between synthetic maps and ground truth

Table S6 Performance of inputs to DSGAN in different datasets.

Dataset	MD			FA		
	T1 + FLAIR	T1	FLAIR	T1 + FLAIR	T1	FLAIR
**PSNR**						
UKB_normal (n=1000)	**31.932** (±1.035)	31.617 (± 0.996)	29.901 (± 0.95)	**29.182** (±0.978)	29.044 (± 0.975)	27.800 (± 0.837)
SCANS (n=120)	**22.710** (±1.983)	22.517 (± 1.984)	20.677 (± 1.014)	23.081 (± 1.354)	**23.246** (±1.578)	22.217 (± 0.763)
RUNDMC (n=502)	26.147 (± 0.983)	**26.459** (±0.923)	24.441 (± 1.040)	25.346 (± 0.848)	**25.364** (±0.921)	23.936 (± 0.694)
PRESERVE (n=105)	24.437 (± 1.409)	**24.937** (±1.356)	22.905 (± 1.665)	24.058 (± 1.153)	**24.195** (±1.042)	23.256 (± 1.742)
NETWORKS (n=26)	24.984 (± 1.359)	**25.305** (±1.321)	23.936 (± 1.254)	**24.296** (±0.916)	24.233 (± 0.964)	23.810 (± 0.888)
NETWORKS(F)	25.074 (± 1.288)	**25.309** (±1.159)	23.726 (± 1.094)	**23.249** (±1.070)	23.105 (± 1.115)	22.779 (± 0.978)
**RMSE**						
UKB_normal (n=1000)	**0.165** (±0.025)	0.171 (± 0.024)	0.211 (± 0.025)	**0.200** (±0.024)	0.204 (± 0.024)	0.235 (± 0.021)
SCANS (n=120)	**0.366** (±0.079)	0.374 (± 0.081)	0.455 (± 0.04)	0.520 (± 0.081)	**0.513** (±0.093)	0.570 (± 0.044)
RUNDMC (n=502)	0.281 (± 0.026)	**0.271** (±0.028)	0.342 (± 0.02)	**0.354** (±0.036)	**0.354** (±0.040)	0.417 (± 0.035)
PRESERVE (n=105)	0.306 (± 0.042)	**0.288** (±0.038)	0.367 (± 0.063)	0.433 (± 0.056)	**0.425** (±0.047)	0.481 (± 0.109)
NETWORKS (n=26)	0.336 (± 0.039)	**0.324** (±0.038)	0.379 (± 0.036)	**0.380** (±0.044)	0.383 (± 0.047)	0.402 (± 0.047)
NETWORKS(F)	0.339 (± 0.027)	**0.330** (±0.025)	0.397 (± 0.025)	**0.376** (±0.026)	0.383 (± 0.027)	0.397 (± 0.028)
**SSIM**						
UKB_normal (n=1000)	**0.971** (±0.007)	**0.971** (±0.007)	0.957 (± 0.007)	**0.906** (±0.014)	0.903 (± 0.014)	0.872 (± 0.016)
SCANS (n=120)	**0.811** (± 0.059)	**0.818** (±0.062)	0.746 (± 0.032)	0.752 (± 0.049)	**0.766** (±0.056)	0.672 (± 0.040)
RUNDMC (n=502)	0.896 (± 0.017)	**0.908** (±0.017)	0.851 (± 0.021)	0.817 (± 0.023)	**0.821** (±0.025)	0.725 (± 0.031)

PRESERVE (n=105)	0.903 (± 0.022)	**0.912** (±0.019)	0.852 (± 0.058)	0.803 (± 0.033)	**0.810** (±0.029)	0.718 (± 0.098)
NETWORKS (n=26)	0.884 (± 0.023)	**0.892** (±0.021)	0.859 (± 0.024)	0.815 (± 0.023)	**0.820** (±0.022)	0.771 (± 0.027)
NETWORKS(F)	0.882 (± 0.021)	**0.888** (±0.018)	0.853 (± 0.022)	0.803 (± 0.024)	**0.805** (±0.024)	0.751 (± 0.026)

Mean (standard deviation) was displayed in each cell. Bold text highlights the model with the highest performance in each dataset. NETWORKS(F) indicates the follow up dataset in the NETWORKS study. This table displayed the RMSE of synthesizing the MD images that had been multiplied by 100. Abbreviations: PSNR: peak signal to noise ratio, RMSE: root mean square error, SSIM: structural similarity index measure.

Table S7 Correlation of the synthetic FA/MD-derived metrics with the ground truth in the external validation datasets.

Metric	UKB_normal (n=1000)	SCANS (n=120)	RUNDMC (n=502)	PRESERVE (n=105)	NETWORKS (n=26)
median FA whole-brain	0.833	0.845	0.800	0.828	0.729
median FA All WM	0.748	0.773	0.828	0.785	0.912
median FA WMH	0.922	0.719	0.870	0.724	0.919
median FA NAWM	0.748	0.736	0.820	0.766	0.895
median MD whole-brain	0.757	0.927	0.907	0.818	0.674
median MD All WM	0.458	0.446	0.444	0.334	0.571
median MD WMH	0.927	0.715	0.828	0.874	0.847
median MD NAWM	0.458	0.298	0.403	0.216	0.367
PSMD	0.555	0.915	0.864	0.914	0.849

Table S8 Visual rating of synthetic MD maps compared with the ground truth. Fractions of synthetic MD maps with a visual grading of 1 were shown. None of the synthetic MD maps had a visual grading of 2 in any category. Unless otherwise stated, synthetic maps have worse visual rating than ground truth.

Dataset	Artefact	Contrast	Presence	Absence	Sharpness
SCANS (n=12)	0.08	0.08	0.00	0.00	0.58
RUNDMC (n=50)	0.08	0.00	0.00	0.00	0.83†
PRESERVE (n=11)	0.45	0.36	0.00	0.00	0.27
NETWORKS (n=6)	0.17	0.17	0.00	0.00	0.83

†All synthetic maps in RUNDMC have higher sharpness than the ground truth.

Table S9 Correlations of FA/MD-derived metrics between the first and second scans in the NETWORKS study (n=14).

Metric	Ground truth	Synthetic
median FA whole-brain	0.933	0.994
median FA All WM	0.946	0.974
median FA WMH	0.935	0.961
median FA NAWM	0.946	0.983
median MD whole-brain	0.925	0.991
median MD All WM	0.942	0.933
median MD WMH	0.933	0.927
median MD NAWM	0.902	0.957
PSMD	0.993	0.996

Abbreviations: WM: white matter, WMH: white matter hyperintensity region, NAWM: normal appearing white matter, i.e., white matter region without WMH.

Table S10 Correlation of the synthetic FA/MD-derived metrics and the ground truth metrics with executive function.

Metric	SCANS (n=120)		RUNDMC (n=502)		PRESERVE (n=104)	
	Ground truth	Synthetic	Ground truth	Synthetic	Ground truth	Synthetic
WMH volume (mL)	-0.078		-0.243 *		-0.234 *	
TBV (mL)	0.213 .		0.246 .		0.026	
median FA whole-brain	0.306 *	0.278 *	0.293 .	0.322 *	0.248 **	0.172 *
median FA All WM	0.248 *	0.330 **	0.323 **	0.283 *	**0.363** **	**0.276** **
median FA WMH	0.236 **	0.228 **	0.077	0.075	0.254 *	0.098
median FA NAWM	0.235 *	0.321 **	0.318 **	0.274 *	0.354 **	0.267 **
median MD whole-brain	**-0.385** **	**-0.345** **	**-0.404** **	**-0.344** **	-0.244 **	-0.279 **
median MD AllWM	-0.227 *	-0.044	-0.370 **	-0.014	-0.299 **	-0.164 .
median MD WMH	-0.193 .	-0.077	-0.133	-0.137	-0.198 .	-0.055
median MD NAWM	-0.218 *	-0.002	-0.365 **	0.011 .	-0.276 **	-0.12
PSMD	-0.250 *	-0.230 *	-0.179	-0.166	-0.321 **	-0.243 **

P values were labelled as: ***: <0.001, **: 0.001-0.01, *: 0.01-0.05, .: 0.05-0.1. Bold text highlights the metrics achieving the highest correlation in each dataset. Abbreviations: TBV: total brain volume, FA: fractional anisotropy, MD: mean diffusivity, All WM: all white matter, WMH: white matter hyperintensity region, NAWM: normal-appearing white matter, PSMD:.peak width of skeletonized mean diffusivity.

Table S11 Correlation of the synthetic FA/MD-derived metrics and the ground truth metrics with processing speed.

**Metric**	**SCANS (n=120)**		**RUNDMC (n=502)**		**PRESERVE (n=104)**	
	Ground truth	Synthetic	Ground truth	Synthetic	Ground truth	Synthetic
WMH volume (mL)	-0.156 *		-0.290 ***		-0.230 *	
TBV (mL)	0.228 *		0.332 **		0.077 .	
median FA whole-brain	0.380 ***	0.293 **	0.399 ***	0.439 ***	0.166 *	0.148 **
median FA All WM	0.242 **	0.340 ***	0.409 ***	0.358 ***	**0.375** ***	**0.374** ***
median FA WMH	0.227 **	0.197 **	0.126 *	0.1	0.317 **	0.155 .
median FA NAWM	0.227 **	0.332 ***	0.404 ***	0.349 ***	0.367 ***	0.373 ***
median MD whole-brain	**-0.452** ***	**-0.399** ***	**-0.515** ***	**-0.463** ***	-0.136 *	-0.222 ***
median MD AllWM	-0.255 **	-0.105	-0.453 ***	-0.078	-0.262 **	-0.326 ***
median MD WMH	-0.211 *	-0.105	-0.253 *	-0.24	-0.186 .	-0.029
median MD NAWM	-0.248 *	-0.063	-0.448 ***	-0.051	-0.243 **	-0.306 **
PSMD	-0.308 **	-0.292 **	-0.412 ***	-0.392 ***	-0.295 ***	-0.172 **

P values were labelled as: ***: <0.001, **: 0.001-0.01, *: 0.01-0.05, .: 0.05-0.1. Bold text highlights the metrics achieving the highest correlation in each dataset. Abbreviations: TBV: total brain volume, FA: fractional anisotropy, MD: mean diffusivity, All WM: all white matter, WMH: white matter in WMH regions, NAWM: normal-appearing white matter, PSMD:.peak width of skeletonized mean diffusivity..

Table S12 Correlation of the synthetic FA/MD-derived metrics and the ground truth metrics with cognition in the NETWORKS study (n=23).

**Metric**	**Executive function**		**Global cognition**		**Processing speed**	
	Ground truth	Synthetic	Ground truth	Synthetic	Ground truth	Synthetic
WMH volume (mL)	0.004		0.023		-0.142	
TBV (mL)	0.290		0.320		0.481	
median FA whole-brain	0.326	0.262	0.351	0.274	**0.574 ***	0.450
median FA All WM	0.345	0.398	0.335	0.365	0.391 *	0.408 *
median FA WMH	-0.008	-0.002	-0.041	-0.105	0.072	0.051
median FA NAWM	**0.394**	**0.476**	**0.386**	**0.451**	0.420 *	0.464 *
median MD whole-brain	-0.228	-0.054	-0.309	-0.152	-0.357	**-0.528 ***
median MD AllWM	-0.216	0.158	-0.295	0.069	-0.168	-0.264 .
median MD WMH	0.028	0.256	-0.055	0.237	-0.216	-0.150
median MD NAWM	-0.235	0.194	-0.333	0.093	-0.105	-0.210
PSMD	-0.222	-0.128	-0.244	-0.176	-0.495 *	-0.385 *

P values were labelled as: ***: <0.001, **: 0.001-0.01, *: 0.01-0.05, .: 0.05-0.1. Bold text highlights the metrics achieving the highest correlation in each dataset. Abbreviations: TBV: total brain volume, FA: fractional anisotropy, MD: mean diffusivity, All WM: all white matter, WMH: white matter in WMH regions, NAWM: normal-appearing white matter, PSMD:.peak width of skeletonized mean diffusivity..

Table S13 Mediation effect of CSVD imaging markers on the association between FA/MD-based metrics and cognition in the RUNDMC cohort (n=502). The coefficients (β) and p-values of the indirect effect of each CSVD imaging marker on the association between each FA/MD-based metric and each cognition marker were shown. The last column shows the p-value of the comparison of the indirect effects between ground truth and synthetic FA/MD-based metrics.

**Cognition**	**Metrics (whole-brain)**	**Ground truth**		**Synthetic**		***P***
		β (95% CI)	*P*	β (95% CI)	*P*	
WMH volume						
Global cognition	Median FA	0.007 (-0.000-0.021)	0.133	**0.018 (0.004-0.042)**	<0.001	0.234
	Median MD	-0.019 (-0.053-0.009)	0.500	-0.018 (-0.052-0.018)	0.436	0.488
Executive function	Median FA	0.006 (-0.000-0.016)	0.133	0.017 (0.004-0.034)	<0.001	0.176
	Median MD	-0.025 (-0.062-0.003)	0.133	-0.026 (-0.072-0.008)	0.240	0.489
Processing speed	Median FA	**0.008 (0.001-0.019)**	<0.001	**0.022 (0.012-0.037)**	<0.001	0.102
	Median MD	**-0.029 (-0.068--0.009)**	<0.001	**-0.030 (-0.072--0.010)**	<0.001	0.487
Lacune counts						
Global cognition	Median FA	**0.020 (0.008-0.039)**	<0.001	**0.025 (0.008-0.043)**	<0.001	0.384
	Median MD	**-0.031 (-0.051--0.006)**	<0.001	**-0.032 (-0.053--0.004)**	<0.001	0.483
Executive function	Median FA	**0.013 (0.006-0.026)**	<0.001	**0.017 (0.006-0.030)**	<0.001	0.361
	Median MD	**-0.020 (-0.038--0.004)**	<0.001	**-0.021 (-0.041--0.004)**	<0.001	0.478
Processing speed	Median FA	**0.024 (0.016-0.039)**	<0.001	**0.029 (0.021-0.048)**	<0.001	0.348
	Median MD	**-0.040 (-0.081--0.024)**	<0.001	**-0.042 (-0.085--0.019)**	<0.001	0.475
TBV						
Global cognition	Median FA	**0.035 (0.010-0.056)**	<0.001	**0.034 (0.008-0.059)**	<0.001	0.484
	Median MD	-0.016 (-0.039-0.027)	0.84	-0.019 (-0.045-0.024)	0.686	0.465
Executive function	Median FA	0.019 (-0.017-0.043)	0.840	0.025 (-0.019-0.048)	0.840	0.427
	Median MD	-0.007 (-0.027-0.037)	1.000	-0.008 (-0.037-0.034)	0.982	0.488
Processing speed	Median FA	0.033 (-0.000-0.068)	0.300	0.029 (-0.009-0.074)	0.300	0.459
	Median MD	-0.016 (-0.066-0.022)	0.600	-0.020 (-0.066-0.020)	0.600	0.464

Bold text indicates that an imaging marker significantly mediates the association between a cognitive marker and a FA/MD-based metric. Abbreviations: CI: confidence interval, TBV: total brain volume, FA: fractional anisotropy, MD: mean diffusivity.

Table S14 C-index in predicting dementia onset by different metrics in multivariate Cox Proportional Hazard models including MRI metric, age, sex and years of education.

**Metric**	**SCANS (n=120)**		**RUNDMC (n=502)**	
	Ground truth	Synthetic	Ground truth	Synthetic
WMH volume (mL)	0.738 *		0.807	
TBV (mL)	0.773 **		0.813 *	
median FA whole-brain	0.829 ***	0.807 ***	0.814 *	0.822 **
median FA All WM	0.761 *	0.804 ***	0.827 ***	0.838 ***
median FA WMH	0.730 .	0.71	0.808	0.807
median FA NAWM	0.756 *	0.808 ***	0.827 ***	0.837 ***
median MD whole-brain	**0.834** ***	**0.828** ***	**0.834** ***	**0.845** ***
median MD All WM	0.760 **	0.752 *	0.820 *	0.814 .
median MD WMH	0.729 *	0.712	0.814 *	0.813 **
median MD NAWM	0.757 **	0.741 *	0.818 *	0.812
PSMD	0.755 ***	0.753 ***	0.835 ***	0.832 ***

P values of the hazard ratio of the MRI metric were labelled as: ***: <0.001, **: 0.001-0.01, *: 0.01-0.05, .: 0.05-0.1. Bold text highlights the metrics achieving the highest c-index in each dataset. Abbreviations: TBV: total brain volume, FA: fractional anisotropy, MD: mean diffusivity, All WM: all white matter, AWM: abnormal white matter, NAWM: normal-appearing white matter, PSMD:.peak width of skeletonized mean diffusivity.

Table S15 C-index in predicting dementia onset by different metrics in multivariate Cox Proportional Hazard models including MRI metric, processing speed, executive function and global cognition.

**Metric**	**SCANS (n=120)**		**RUNDMC (n=502)**	
	Ground truth	Synthetic	Ground truth	Synthetic
WMH volume (mL)	0.885		0.810	
TBV (mL)	0.893		0.827 ***	
median FA whole-brain	0.898	0.897	0.831 ***	0.846 ***
median FA All WM	0.883	0.887	0.837 ***	0.847 ***
median FA WMH	0.884	0.894	0.812	0.811
median FA NAWM	0.883	0.892	0.837 ***	0.845 ***
median MD whole-brain	**0.903 .**	**0.903 .**	**0.854** ***	**0.858** ***
median MD All WM	0.886	0.883	0.838 ***	0.821 **
median MD WMH	0.886	0.885	0.829 ***	0.833 ***
median MD NAWM	0.886	0.885	0.834 ***	0.819 **
PSMD	0.888	0.885	0.847 ***	0.842 ***

P values of the hazard ratio of the MRI metric were labelled as: ***: <0.001, **: 0.001-0.01, *: 0.01-0.05, .: 0.05-0.1. Bold text highlights the metrics achieving the highest c-index in each dataset. Abbreviations: TBV: total brain volume, FA: fractional anisotropy, MD: mean diffusivity, All WM: all white matter, AWM: abnormal white matter, NAWM: normal-appearing white matter, PSMD:.peak width of skeletonized mean diffusivity.

Table S16 Performance of different variations of the GAN model on the subset of 100 patients from the validation dataset in UKB_WMH.

Experiment	PSNR	RMSE	SSIM
3D GAN (λ_{NMSE} = 0)	30.599 (± 1.036)	0.013 (± 0.002)	0.965 (± 0.007)
3D GAN (λ_{NMSE} = 10)	30.779 (± 0.993)	0.013 (± 0.002)	0.965 (± 0.006)
3D GAN (λ_{NMSE} = 100)	30.84 (± 1.025)	0.013 (± 0.002)	0.966 (± 0.006)
2D GAN (λ_{NMSE} = 100)	29.618 (± 0.918)	0.015 (± 0.002)	0.946 (± 0.008)
3D GAN + SN (patchGAN)	30.856 (± 1.022)	0.013 (± 0.002)	0.966 (± 0.006)
3D GAN + SN (patchGAN) + FM	**30.873 (± 0.998)**	0.013 (± 0.002)	0.966 (± 0.006)
3D GAN + SN (patchGAN) + attention	30.837 (± 1.047)	0.013 (± 0.002)	0.966 (± 0.006)
3D GAN + SN (patchGAN) + FM + attention	30.829 (± 1.008)	0.013 (± 0.002)	0.966 (± 0.006)

Bold text indicates the model with the highest performance in a particular metric. Abbreviations: λ_{NMSE} : weight of the normalized mean squared error (NMSE) loss, SN: spectral normalization, FM: feature matching, PSNR: peak signal to noise ratio, SSIM: structural similarity index measure, RMSE: root mean squared error.
